# Assessing Antibacterial Properties of Copper Oxide Nanomaterials on Gut-Relevant Bacteria In Vitro: A Multifaceted Approach

**DOI:** 10.3390/nano15141103

**Published:** 2025-07-16

**Authors:** Tia A. Wardlaw, Abdulkader Masri, David M. Brown, Helinor J. Johnston

**Affiliations:** NanoSafety Research Group, School of Engineering and Physical Sciences, Heriot-Watt University, Edinburgh EH14 4AS, UK; tw44@hw.ac.uk (T.A.W.); abd-almassri@hotmail.com (A.M.); d.brown@hw.ac.uk (D.M.B.)

**Keywords:** nanomaterials, antibacterial, copper oxide, in vitro, nanotoxicology, reactive oxygen species

## Abstract

Due to the growth in the application of antibacterial nanomaterials (NMs), there is an increased potential for ingestion by humans. Evidence shows that NMs can induce dysbiosis in the gut microbiota in vivo. However, in vitro investigation of the antibacterial activity of NMs on gut-relevant, commensal bacteria has been neglected, with studies predominantly assessing NM toxicity against pathogenic bacteria. The current study investigates the antibacterial activity of copper oxide (CuO) NMs to *Escherichia coli* K12, *Enterococcus faecalis*, and *Lactobacillus casei* using a combination of approaches and evaluates the importance of reactive oxygen species (ROS) production as a mechanism of toxicity. The impact of CuO NMs (100, 200, and 300 μg/mL) on the growth and viability of bacterial strains was assessed via plate counts, optical density (OD) measurements, well and disc diffusion assays, and live/dead fluorescent imaging. CuO NMs reduced the viability of all bacteria in a concentration-dependent manner in all assays except the diffusion assays. The most sensitive methods were OD measurements and plate counts. The sensitivity of bacterial strains varied depending on the method, but overall, the results suggest that *E. coli* K12 is the most sensitive to CuO NM toxicity. The production of ROS by all bacterial strains was observed via DCFH-DA fluorescent imaging following exposure to CuO NMs (300 μg/mL). Overall, the data suggests that CuO NMs have antibacterial activity against gut-relevant bacteria, with evidence that NM-mediated ROS production may contribute to reductions in bacterial viability. Our findings suggest that the use of a combination of assays provides a robust assessment of the antibacterial properties of ingested NMs, and in particular, it is recommended that plate counts and OD measurements be prioritised in the future when screening the antibacterial properties of NMs.

## 1. Introduction

Nanomaterials (NMs) are defined as having at least one dimension < 100 nm [1]. The physicochemical (PC) properties of NMs (e.g., composition, size, and shape) determine their diverse functions (such as antibacterial activity) and have led to their utilisation in various applications such as pharmaceuticals, electronics, diagnostics, cosmetics, and textiles [2,3,4,5,6,7,8]. The food industry is a leading exploiter of nanotechnology as NMs can be utilised in agriculture, food processing, nutritional supplements, and packaging [9,10]. For example, titanium dioxide (TiO_2_) particles (which have a nano component) can be used as a pigment in foods [10]. Copper (Cu) NMs are used as pesticides, as they exhibit antifungal and antibacterial properties against many crop plant pests [11]. The antibacterial properties of NMs are also exploited in food contact materials. For example, silver (Ag), zinc oxide (ZnO), Cu, and TiO_2_ NMs also function as antimicrobial agents in food packaging [10,12]. In addition, antibacterial NMs can be used in a clinical setting to treat bacterial infections and have been used in wound dressings, implant devices, as antibiotic delivery systems, and in antibacterial vaccines [13,14].

As industries continue to exploit NMs, increased human exposure via ingestion will inevitably occur [15]. For example, NMs can leach from food contact materials (e.g., food packaging) into food, and NMs may contaminate crops when used as pesticides, leading to their accidental ingestion. In addition, inhaled NMs may interact with the gastrointestinal tract (GIT) following mucociliary clearance from the lungs [16,17,18]. NMs may also be unintentionally ingested due to hand-to-mouth contact (e.g., in an occupational setting or due to their use in consumer products (e.g., wood preservatives) [19]. Furthermore, the increased use of NMs in several consumer products can lead to their release into the environment and contamination of drinking water [20,21]. While most existing studies focus on the antibacterial properties and toxicity of Ag NMs, there is comparatively limited research on other antibacterial nanomaterials such as copper oxide (CuO) [22,23,24,25,26]. This study, therefore, focuses on CuO NMs to address this knowledge gap.

Additionally, toxicology research has also primarily examined the effects of NMs following inhalation; however, as antibacterial NMs are likely to be ingested, they may have a detrimental impact on the gut microbiota to cause adverse health outcomes. Of concern is that ingestion of Cu by humans can disrupt the GIT, inducing abdominal pains, vomiting, and diarrhoea [27]. The gut microbiota is a diverse microbial community within the GIT and plays an essential role in maintaining human health [28,29]. Firmicutes and Bacteroidetes account for 90% of the gut microbiota, with Firmicutes encompassing ~200 different genera, namely, *Clostridium*, *Lactobacillus*, *Bacillus*, *Enterococcus*, and *Ruminococcus* [30]. The commensal bacteria within the gut microbiota are critical to specific host functions [30]. Commensals such as *Enterococcus* spp. and *Escherichia coli* (*E. coli*) colonise and regulate the digestive system and modulate immune responses; *Lactobacillus* spp. also enhance gut barrier integrity [30]. There is an indisputable link between dysbiosis of the gut microbiota and disease pathogenesis, namely, diabetes, liver disease, and obesity, among others [28,29].

Research has shown numerous NMs to have broad-spectrum antibacterial activity against both Gram-negative and Gram-positive bacteria. Ag and ZnO NMs are commonly assessed and have been observed to have antibacterial activity against many bacterial strains, such as *E. coli*, *Bacillus subtilis* (*B. subtilis*), *Pseudomonas aeruginosa* (*P. aeruginosa*), *Staphylococcus aureus* (*S. aureus*), *Salmonella enterica*, *Listeria monocytogenes*, and *Vibrio cholera* [31,32,33,34,35,36,37,38,39,40]. Studies have also reported Ag and ZnO NMs to have antibacterial behaviour against antimicrobial-resistant bacterial strains, including methicillin-resistant *S. aureus* (MRSA) and *E. coli* [41,42,43].

The assessment of antibacterial NMs on the gut microbiota in vivo has increased over recent years; for instance, Zhou et al. (2021) [44] used an in vitro approach to assess the effect of ZnO NMs on the microbial composition of human faecal samples. Following NM treatment, the relative abundance of the genera Firmicutes decreased and Actinobacteria increased with a reduction in bacterial viability overall. An in vivo study orally administered Ag NMs to mice, and 16s rRNA sequencing identified an alteration in the Bacteroidetes/Firmicutes ratio in the gut of NM-treated mice. Bacteroidetes increased from 73% to 85%, and Firmicutes decreased from 26% to 15%, both compared to the control. Notably, *Lactobacillus* in the gut also decreased following NM treatment [45]. Van den Brule et al. (2016) [46] found mice that were orally exposed to food supplemented with Ag NMs over 28 days displayed an increased ratio of Bacteroidetes to Firmicutes in the gut bacterial community. Other NMs have also received attention for their antibacterial properties, including CuO and TiO_2_, albeit to a lesser extent [11,13,22,23,47,48,49]. One in vivo study found the composition of the gut microbiota of zebrafish was altered following dietary intake of Ag and CuO NMs [24]. Similarly, Swart et al. (2020) [25] showed via DNA isolation, PCR, and 16s rRNA sequencing that earthworms treated with CuO NMs for 28 days presented a shift in the bacterial community of the gut. However, not all studies have found antibacterial NM to induce dysbiosis of commensal gut bacteria. For example, Sizentsov et al. (2018) [26] assessed the effects of Cu and CuO NMs on the probiotic strains *Enterobacterium*, *Lactobacillus*, and *Enterococcus* isolated from the pure intestinal cultures from broiler chickens. No significant alteration in the probiotic bacterial strains at low concentrations of NM exposure was observed, which is postulated to be due to the slow release of Cu ions [26]. Additionally, Pietroiusti et al. (2016) [50] found Cu complexed to chitosan NMs to have antibacterial activity against pathogenic *Salmonella* while not affecting commensal *Lactobacillus.* Wilding et al. (2016) [51] assessed microbial communities in mice via 16s rRNA sequencing 28 days post oral exposure to Ag NMs (20 and 110 nm in size and coated with PVP and citrate). The Ag NMs induced no changes in the structure, diversity, or composition of the gut microbiota. Due to conflicting findings across studies and a lack of assessments on commensal bacterial strains, it is necessary to further evaluate the potential impact of NMs on the gut microbiota.

There are many benefits to using in vitro studies to assess the antibacterial activity of NMs. They allow for the direct evaluation of the potential bactericidal or bacteriostatic effects of NMs, offer enhanced control over experimental conditions, and reduce ethical concerns. There are numerous methods for the assessment of the antibacterial properties of NMs, and some of the most common include disc and well diffusion, microtiter plate assays (e.g., qPCR, live/dead assay, and Alamar Blue assay), optical density (OD) measurements, fluorescent microscopy, and colony plate counts [38,52]. Using a battery of approaches provides a more robust toxicity assessment and allows for a comparison of the sensitivity of different methods as they assess the bacterial response via different mechanisms. In a recent in vitro study, CuO NMs were found to act as an antibacterial agent to a varying degree against pathogenic bacteria (*B. subtilis* > *E. coli* > *S. aureus* > *P. aeruginosa*) [22]. Masri et al. (2022) [22] observed that the methods employed to assess the antibacterial activity of CuO NMs varied in their sensitivity, finding plate counts (time kill assay) and OD measurements to be the most effective. Additionally, Masri et al. (2022) [22] used live/dead fluorescent imaging to assess the antibacterial activity of CuO NM, as it is sensitive and allows for a visual indication of bacterial viability. Similarly, Hossain et al. (2019) [53] utilised a multifaceted approach to assess the antibacterial activity of Ag NMs against both pathogenic and non-pathogenic bacteria in vitro. Plate counts, disc diffusion assays, and OD readings demonstrated Ag NMs to have antibacterial activity against all bacterial strains, with the highest activity against pathogenic *S. aureus.* Sirelkhatim et al. (2015) [52] investigated the antibacterial effect of ZnO NMs on *E. coli* via 24-h OD measurements, finding bacteriostatic inhibition to increase in a concentration-dependent manner. The lack of standard protocols available to assess antibacterial NMs has led to studies varying drastically in their experimental design; for example, NM concentrations, exposure duration, and experimental procedure (e.g., protocol, growth media, and bacterial strain) can influence antibacterial potency [22,26,54]. This makes it challenging to compare results between studies and may explain why there are such conflicting findings across studies.

In vitro techniques also allow for the assessment of the potential toxic mechanisms that underpin the antibacterial activity of NMs. The currently hypothesised molecular mechanism by which NMs elicit their antibacterial properties includes oxidative stress, the release of toxic ions, and the direct interaction between NMs and the bacterial cell surface [23,34,36,53,55,56]. Numerous studies have highlighted the importance of oxidative stress as a main antibacterial mechanism of action (MOA) of NMs [23,32]. It is hypothesised that ROS and free radicals reduce bacterial cell viability by stimulating a loss of membrane integrity and by promoting pore formation of the bacterial membrane, causing cell lysis [57,58,59]. Studies suggest ROS generation is directly promoted by NM interactions with bacteria [23,60,61]. For example, Li et al. (2012) [23] observed that *E. coli* K12 viability (using plate counts) reduced to varying degrees depending on NM treatment (CuO > TiO_2_ > ZnO > Al_2_O_3_ > SiO_2_ > Fe_2_O_3_ > CeO_2_) and demonstrated a correlation between antibacterial activity and increased ROS generation [23]. Kim et al. (2007) [32] reported that Ag NMs generated free radicals, which inhibited the growth of *E. coli* and moderately reduced the growth of *S. aureus*. Zheng et al. (2017) [55] also assessed intracellular ROS production within *Staphylococcus epidermidis* (*S. epidermidis*), *S. aureus*, *B. subtilis*, and *P. aeruginosa* following gold (Au) NM treatment via the DCFH-DA assay, observing a correlation between the antimicrobial effect and ROS production. Overall, while many approaches are available to assess antibacterial NMs in vitro, there are limitations to their use. In vitro methods are still to be standardised, and their suitability and sensitivity for testing NMs still require evaluation [22].

**Figure 1:**
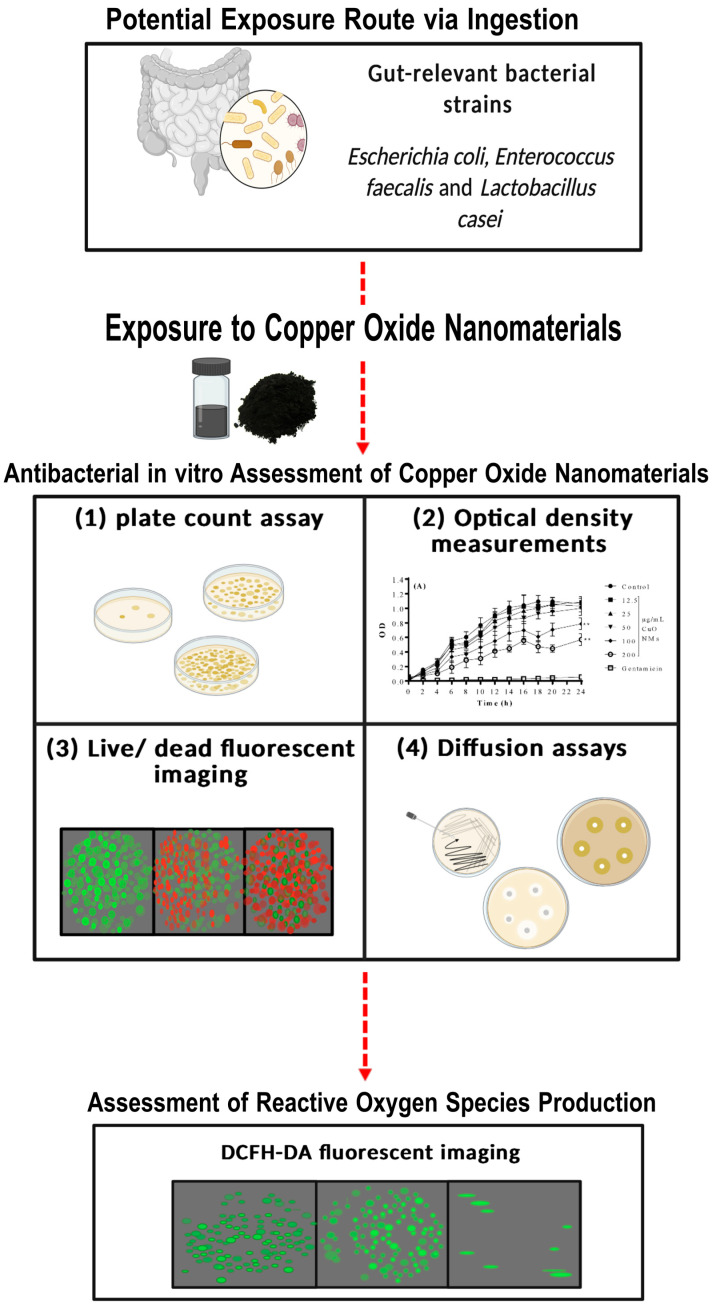
Schematic illustration of the experimental approach used within this study. Gut-relevant bacterial strains were exposed to CuO NMs, and bacterial viability was assessed using a combination of approaches. (1) Plate count assay, (2) optical density measurements, (3) live/dead fluorescent imaging, and (4) diffusion assays. Following this, fluorescent imaging employing a DCFH-DA probe was used to visualise the change in ROS levels in the bacterial cells after CuO NM treatment. Created in BioRender (2025, Edinburgh, UK).

The primary aim of the current study was to evaluate the antibacterial activity of CuO NMs to gut-relevant bacterial strains using a multifaceted approach. More specifically, the antibacterial activity of CuO NMs was investigated via application of a battery of tests, including viability (plate count) assays, optical density (OD) measurements, well and disc diffusion assays, and live/dead fluorescent imaging. By comparing the sensitivity of different assays, we aim to identify which approaches should be prioritised in the future when investigating the antibacterial activity of NMs in vitro. In addition, we aim to evaluate the contribution of ROS generation to the antibacterial properties of CuO NMs to improve the understanding of the mechanisms that drive antibacterial activity. The antibacterial activity of CuO NMs against key gut bacterial (commensal) strains was assessed, namely *Escherichia coli K12* (*E. coli K12*), *Enterococcus faecalis* (*E. faecalis*), and *Lactobacillus casei* (*L. casei*) (Figure 1). *E. coli* was selected as a representative Gram-negative human gut commensal bacterium that constitutes 0.1–5% of the bacterial community [28]. *E. faecalis* is Gram-positive and was selected as another commensal bacterium of the human gut, which is known to be an opportunistic pathogen [62]. *L. casei* was selected as it is a probiotic commensal bacterium that is integral to host health [26]. By examining both Gram-positive and negative strains, we aim to identify whether Gram-positive or negative bacteria vary in their sensitivity to NM toxicity, as previous studies have found the antibacterial effect of NMs on different bacterial strains varies, with previous studies generally observing Gram-negative bacteria being more susceptible to antibacterial NMs than Gram-positive strains [56,63].We hypothesise that CuO NMs will induce an antibacterial response and will reduce bacterial cell viability within all bacteria tested, with Gram-negative *E. coli* exhibiting greater sensitivity to NM toxicity. Furthermore, we anticipate that CuO NMs will elevate ROS levels in bacteria to reduce bacterial cell viability.

## 2. Materials and Methods

### 2.1. CuO Nanomaterial Characterisation

CuO NMs were obtained from PlasmaChem, Gmbh (Berlin, Germany). The manufacturer information data sheet reported the NMs to range in size from 15 to 20 nm, have a specific surface area of 47 m^2^/g, and have a density of 6.3 g/cm^3^ employing the Brunauer–Emmett–Teller (BET) method [19]. Comprehensive characterisation of the CuO NMs used in this study has already been reported in previous research [64]. More specifically, the size and morphology of the CuO NMs were characterised by Gosens et al. (2016) [64,65]. Briefly, CuO NMs were analysed via transmission electron microscopy (TEM) (Hillsboro, Oregon, USA) and X-ray diffraction (XRD) (Philips, Eindhoven, Netherlands), with a primary particle size measured of 10 nm and 9.3 nm, respectively. Gosens et al. (2016) [64] and Pantano et al., 2018 [65] present the TEM images of the pristine CuO NMs in their study and demonstrate that the CuO particles agglomerate. Cu dissolution has been assessed previously via inductively coupled plasma optical emission spectrometry (ICP-OES) Faenza, Italy) and was measured at <1.5% at pH 7.4 and ~62% at pH 4.5 in Gamble’s solution at 1 and 24 h [64,66]. In the present study, independent characterisation of NM PC properties was performed in sterile distilled water (50 μg/mL) via Dynamic Light Scattering (DLS, Malvern Zeta sizer Nano series) to assess the hydrodynamic diameter, zeta (ζ) potential, and polydispersity index (PDI) of a CuO NM suspension. Measurement of the hydrodynamic diameter reflects the size of nanomaterials in suspension. The PDI is a dimensionless measure of the particle size distribution, and the zeta (ζ) potential reflects the electrostatic surface charge of particles in suspension and is a key indicator of colloidal stability.

### 2.2. CuO Nanomaterial Preparation

CuO NM stock suspensions were prepared at a concentration of 1 mg/mL using sterile distilled water and bath sonicated (max capacity 1.5 L, ultrasonic power 36 W) for 15 min [22]. Stock suspensions were then diluted in distilled water, Mueller-Hinton broth (MHB), or Tryptone soya broth (TSB) to a concentration range of 100, 200, and 300 μg/mL. NM concentration selection was informed by existing studies [22,67].

### 2.3. Bacterial Models and Culture Conditions

*Escherichia coli* (*E. coli*) K12, *Enterococcus faecalis* (*E. faecalis*), and *Lactobacillus casei* (*L. casei*) were utilised as model organisms in this study. Stock cultures of *E. coli* K12 (NCTC 14582), *E. faecalis* (NCTC 775), and *L. casei* (NCTC 13764) were obtained from the National Collection of Type Cultures (NCTC). *E. coli* K12 and *E. faecalis* were cultured and grown in MHB and Mueller–Hinton agar (MHA). *L. casei* was cultured and grown in TSB and Mann, Rogosa, and Sharp agar (MRSA). The bacteria were cultured on a shaking incubator (250 rpm) at 37 °C overnight in aerobic conditions. MHA and MRSA (<20 mL) were poured into Petri dishes (100 mm D × 15 mm H) under sterile conditions (airflow hood) and left to dry. To ensure uniformity of bacteria within all experiments, standardisation of bacteria was performed. Evaluation of the number of CFU for each strain was completed by measuring turbidity at 600 nm using a UV/visible spectrophotometer (Edinburgh, UK). The bacterial suspensions were diluted to a final concentration of ~5.0 × 10^5^ CFU/10 μL in specific culture media (MHB or TSB) for all assays [22]. Culture media was utilised as a negative control and 10 μg/mL gentamicin as a positive control for all experiments.

### 2.4. Time Kill Assay (Plate Count)

Each bacterial inoculum (10 μL) was added to 190 μL of CuO NM suspensions (100–300 μg/mL) or the positive or negative controls in 1.5 mL Eppendorf tubes. The bacterial samples were vortexed and then incubated at 37 °C in a shaking incubator (250 rpm) for 2 h. For each bacterial strain, 10 μL of the samples was then diluted via 10-fold serial dilution using sterile distilled water. Following this, 10 μL of each dilution for *E. coli* K12 and *E. faecalis* were plated onto the surface of MHA plates, and 10 μL of each dilution for *L. casei* were plated onto MRSA plates and incubated overnight at 37 °C. The viable bacteria colonies were then counted by CFUs [22].

### 2.5. Optical Density Measurement

Ten microlitres (10 μL) of each bacterial inoculum was exposed to 190 μL of CuO NM suspensions (100–300 μg/mL) or the positive or negative control in each well of a 96-well plate. To compensate for potential NM interference, NMs in specific culture media (100–300 μg/mL) in the absence of bacteria were also included as a control. The plate was then incubated at 37 °C for 24 h. To quantify the OD, absorption was measured at 600 nm at 2 h intervals for 24 h via UV–Vis spectroscopy (microplate reader) (FLUOstar Omega (version 5.10), BMG LABTECH, Aylesbury, UK). To account for the potential NM interference, OD values of NM interference control were subtracted from OD values in the presence of bacteria. This is essential for appropriate data interpretation, as the optical properties of NMs are known to interfere with results [68].The data generated a bacterial growth curve for all bacteria strains [67,69].

### 2.6. Diffusion Assay (Well and Disc)

Diffusion assays were employed to assess the inhibition of bacterial growth via measurement of the zone of inhibition (ZOI) 24 h post-exposure. For both assays, MHA or MRSA Petri dishes were seeded with 100 μL of each bacterial suspension using a sterile cotton spreader. The plates were then dried at room temperature (RT) for 10–20 min. For the well diffusion assay, five wells (6 mm in diameter) were made in the agar plates via a sterile steel-core borer, and the well bases were sealed with 5 μL of molten agar. The wells were then loaded with 50 μL of CuO NMs (100–300 μg/mL) or the positive or negative control. The plates were then dried at RT for 30 min, followed by a 24 h incubation at 37 °C. The ZOI around each well was measured in mm to calculate the actual ZOI. Six mm was subtracted from the ZOI measurement to account for the diameter of the well [70]. For the disc diffusion (Kirby–Bauer) assay, Whatman filter paper antibiotic discs (6 mm) were gently placed on the agar plate. Following this, 10 μL of CuO NMs (100–300 μg/mL) or the negative or positive control were loaded onto paper discs. The plates were incubated for 24 h at 37 °C. The ZOI around each paper disc was measured in mm, and disc diameters (6 mm) were subtracted to calculate the actual ZOI [71].

### 2.7. Live/Dead Fluorescent Images

A commercially available kit (ThermoFisher (Waltham, MA, USA), LIVE/DEADTMBacLightTM Bacterial Viability Kit, L7012) was employed as per the manufacturer’s instructions to perform the live/dead imaging. In a 15 mL Falcon tube, 1 mL of bacterial cells were exposed to 300 μg/mL CuO NMs, positive or negative controls. Cells were then incubated for 2 h, followed by microscopy staining. Fluorescent microscopy was used to image the cells following staining with the live/dead stain using a Leica DM IRBE CLSM (Edinburgh, UK) (100× magnification).

### 2.8. DCFH-DA Fluorescent Images

The generation of intracellular ROS was qualitatively assessed via fluorescent microscopy. The bacterial strains (1 mL) were incubated in a 15 mL Falcon tube with 300 μg/mL CuO NMs, positive control (10 μM (~3.4 × 10^−5^% *w*/*w*) hydrogen peroxide (H_2_O_2_)), or negative control (untreated cells) in specific culture media for 2 h. Then, the bacterial suspensions were incubated with the 10 mM 2,7-dichlorodihydrofluorescein diacetate (DCFH_2_-DA) probe diluted with methanol for 30 min in the dark. Following this, samples were mounted onto slides, and images were taken at 40× magnification using a Leica DM IRBE CLSM.

### 2.9. Statistical Analysis

Each experiment was performed as three independent replicates, and data are expressed as average ± standard error of the mean (SE). All statistical analyses were conducted with Minitab software [version 18]. Data for all assays were screened for normality using the Anderson–Darling test. Parametric data were analysed using an ANOVA, with a Tukey post hoc test identifying differences between the treatment groups. Non-parametric data were subjected to the Kruskal–Wallis test with a Kruskal–Wallis post hoc test. The results were considered statistically significant with a *p* value of ≤0.05. Details of the statistical analysis performed for each assay are provided in the figure legends for all experiments.

## 3. Results

### 3.1. CuO NM Characterisation

The average hydrodynamic diameter of CuO NMs in sterile distilled water was 201.68 nm ± 4.3. The polydispersity index (PDI) was 0.31 ± 0.08, and the charge of the CuO particles was neutral with a ζ-potential of 0.005 mV ± 0.04. Our CuO NMs exhibited a PDI which indicates moderate polydispersity, which is not uncommon in metal oxide nanomaterial suspensions. These specific CuO NMs were selected to reflect a realistic and representative formulation of commercially available CuO NMs, rather than an idealised or artificially stabilised system. Their moderate PDI and neutral surface charge offer a relevant model for how such particles would potentially behave under practical exposure conditions.

### 3.2. Time Kill (Plate Count) Assay 

**Figure 2:**
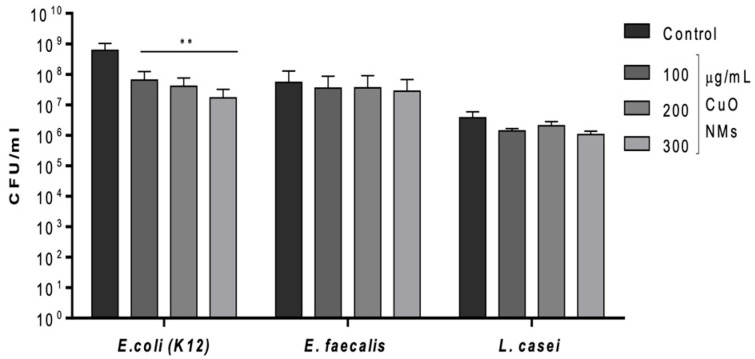
Bacterial growth following 2 h exposure to CuO NMs. *E. coli*, *E. faecalis*, and *L. casei* were treated with CuO NMs at concentrations of 100, 200, and 300 µg/mL (in MHB or TSB). Viable cell counts were measured by culturing bacterial colonies on MHA (*E. coli* and *E. faecalis*) or MRSA (*L. casei*) plates. A negative control (MHB or TSB) and positive control (10 µg/mL gentamicin) were also included. Data are expressed as the mean CFU/mL ± SE (*n* = 3). ANOVA with a post hoc Tukey test was performed with significance indicated by ** = *p* < 0.01 compared to the control.

The viability (plate count) assay found the bacteria to vary in their sensitivity to CuO NMs following a 2 h incubation. CuO NMs induced an antibacterial response in *E. coli* K12 in a concentration-dependent manner (Figure 2). The number of viable *E. coli* K12 was significantly reduced at all tested concentrations compared to the negative control (MHB for *E. coli* K12) (*p* < 0.01) (Figure 2). There was no significant decrease in the viability of *E. faecalis* or *L. casei*, compared to the control (Figure 2). Based on the results of this assay, the toxicity of CuO NMs to the bacteria can be ranked as follows (from most to least sensitive): *E. coli* K12 > *E. faecalis* = *L. casei.* All bacteria exposed to 10 μg/mL of gentamicin exhibited 100% killing. 

### 3.3. OD Measurements

**Figure 3:**
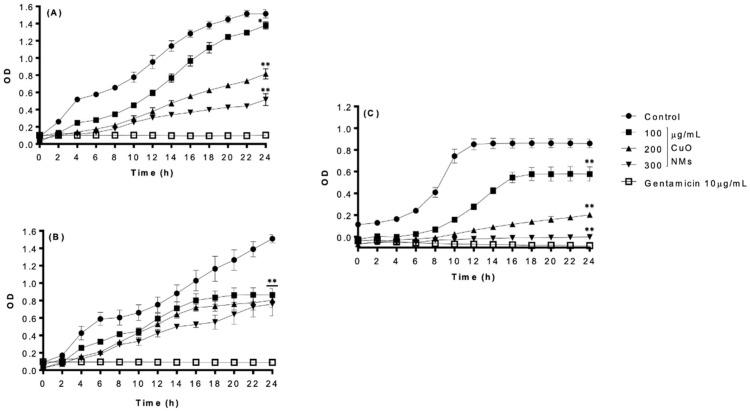
Bacterial growth following exposure to CuO NMs (OD Measurement). Bacterial growth curves of (**A**) *E. coli*, (**B**) *E. faecalis*, and (**C**) *L. casei* following exposure to CuO NMs at concentrations of 100, 200, and 300 µg/mL (in distilled water) for 24 h. Bacteria were also exposed to either 10 µg/mL gentamicin (positive control) or MHB (*E. coli* and *E. faecalis*) or TSB (*L. casei*) (negative control). OD measurements were made at 600 nm. Data are expressed as the mean OD ± SE (n = 3). Kruskal–Wallis or ANOVA with a post hoc Tukey test was performed with significance indicated by ** = *p* < 0.01 compared to the control, with significance indicated by * = *p* < 0.05 and ** = *p* < 0.01 compared to the control at 24 h.

All bacteria exposed to the negative control were observed to have increased OD over time, which is indicative of bacterial growth (Figure 3). CuO NMs exhibited concentration- and time-dependent antibacterial activity against all bacteria, with the strongest growth inhibition observed at 24 h post-exposure (Table 1). Following 24 h of exposure to 300 μg/mL of CuO NMs, *L. casei* displayed the highest sensitivity, while *E. faecalis* showed greater resistance, presenting a 100% reduction and 50% reduction in cell viability, respectively (Figure 3B,C). The inhibition of *E. coli* K12 growth following exposure to 300 μg/mL CuO NM was reduced by 66% when compared to the control (Table 1). Treatment with gentamicin (10 µg/mL) effectively inhibited bacterial growth at all time points (Figure 3).

### 3.4. Diffusion Assays

**Figure 4:**
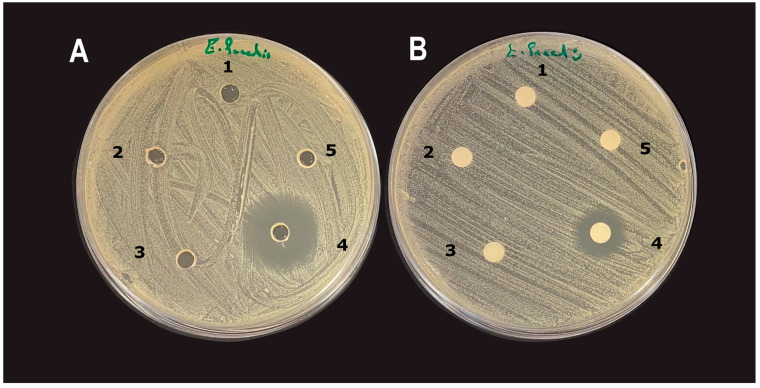
(**A)** Well diffusion assay and (**B**) disc diffusion assay inhibition zones of CuO NMs and gentamicin (10 µg/mL) on the growth of *E. faecalis*. (1) CuO NM 100 µg/mL; (2) CuO NM 200 µg/mL; (3) CuO NM 300 µg/mL; (4) gentamicin 10 µg/mL; (5) control (distilled water).

Interestingly, both the well and disc diffusion assays found CuO NMs to have no impact on bacterial growth, regardless of concentration or bacterial strain. Only gentamicin (10 µg/mL) exhibited inhibition of bacterial growth in both the well and disc diffusion assays (Figure 4). Disc diffusion inhibition zones measured were *E. coli* k12 = 4 ± 1.8 mm, *E. faecalis* = 7.5 ± 2 mm, and *L. casei* = 0.3 ± 1.6 mm. Well diffusion inhibition zones were *E. coli* K12 = 14 ± 1.4 mm, *E. faecalis* = 17 ± 2 mm, and *L. casei* = 4.6 ± 2 mm.

### 3.5. Live/Dead Fluorescent Microscopy

**Figure 5:**
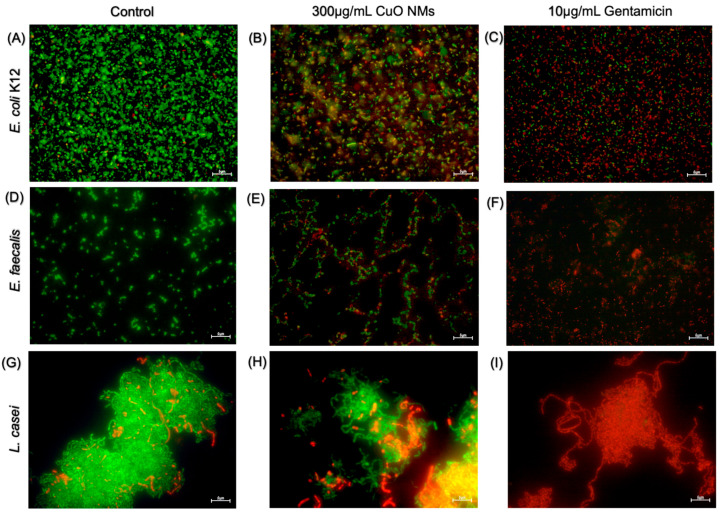
Imaging of live and dead cells following CuO NM exposure. Representative microscopic images of bacteria exposed to MHB or TSB (negative control), gentamicin 10 μg/mL (positive control), or CuO NMs for 2 h. *E. coli* K12; (**A**) Untreated control (MHB), (**B**) CuO NM 300 µg/mL, (**C**) gentamicin 10 μg/mL. *E. faecalis*; (**D**) untreated control (MHB), (**E**) CuO NM 300 µg/mL, (**F**) gentamicin 10 μg/mL. *L. casei*; (**G**) untreated control (TSB), (**H**) CuO NM 300 µg/mL, (**I**) gentamicin 10 μg/mL. Bacteria were stained using the live/dead stain to visualise dead (red) and viable (green) cells. Bacterial cells were imaged at 100× magnification using a Leica DM IRBE CLSM. The scale bar is 5 μm.

Fluorescent microscopy was performed to visualise the impact of CuO NMs on bacterial viability using the live/dead assay. The majority of control cells for all bacteria strains were viable (green), with very few or no dead (red) cells (Figure 5A,D,G). The bacterial cells exposed to gentamicin (10 μg/mL) in all bacterial models were almost all dead (Figure 5C,F,I). Following exposure to CuO NMs at a concentration of 300 μg/mL, a decrease in cell viability was observed for all bacteria, with an increased proportion of dead cells observed in all bacterial strains when compared to the control (Figure 5B,E,H). The images suggest *E. coli* K12 appears to be the most sensitive to the toxicity of the CuO NMs, as the highest proportion of dead cells is observed (Figure 5B).

### 3.6. DCFH-DA Fluorescent Microscopy

**Figure 6:**
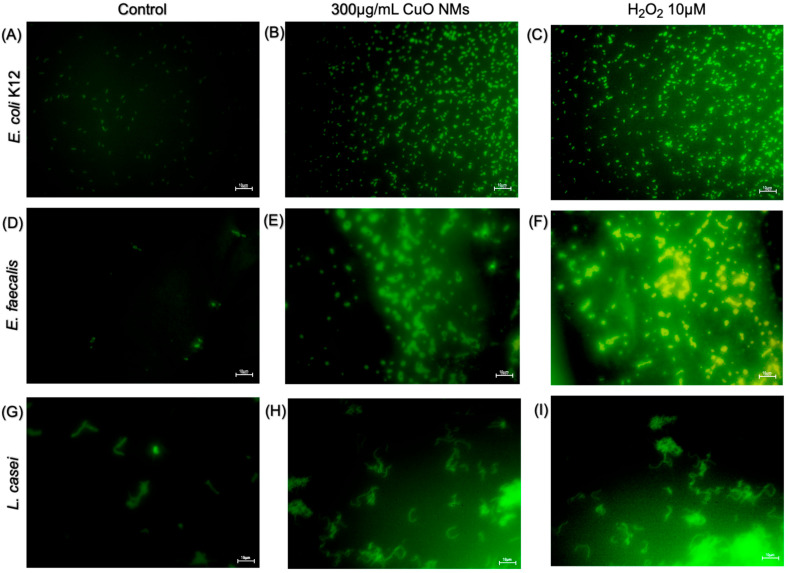
Imaging of intracellular ROS generation by bacterial cells following CuO NM exposure. Representative microscopic images of bacteria exposed to MHB or TSB (negative control), H_2_O_2_ (10 μM) (positive control), or CuO NMs (300 µg/mL) for 2 h. *E. coli* K12; (**A**) untreated control (MHB), (**B**) CuO NM, (**C**) H_2_O_2_ *E. faecalis*; (**D**) untreated control (MHB), (**E**) CuO NM 300 µg/mL, (**F**) H_2_O_2_ 10 μM. *L. casei*; (**G**) untreated control (TSB), (**H**) CuO NM 300 µg/mL, (**I**) H_2_O_2_ 10 μM. Bacteria were stained using the DCFH-DA probe to visualise ROS generation (green) in bacterial cells. Bacterial cells were imaged at 40× magnification using a Leica DM IRBE CLSM. The scale bar is 10 μm.

The DCFH-DA fluorescent probe was used to visualise the impact of CuO NMs on intracellular ROS production. DCFH-DA is taken up by the cell via the cellular membrane. Following this, hydrolysis via cellular esterase converts the probe to DCFH. Intracellular ROS then oxidise DCFH, producing the highly fluorescent DCF molecule [63]. Therefore, the intensity of the DCF (green) fluorescence provides a visual indication of ROS within the bacterial cells. The majority of control cells for all bacteria models displayed weak to no fluorescence (Figure 6A,D,G). The bacterial cells exposed to the positive control (10 μM H_2_O_2_) in all bacterial models demonstrated the highest fluorescent intensity, thus inducing the greatest production of ROS (Figure 6C,F,I). The fluorescence of bacterial cells following exposure to 300 μg/mL CuO NMs appeared to increase compared to the control; however, not to the same extent as H_2_O_2_-treated cells (Figure 6B,E,H).

## 4. Discussion

### 4.1. Impact of CuO NMs on Commensal Gut Bacteria

It is well established that CuO NMs have antibacterial activity and are commonly exploited for this attribute [9,11]. Although increased accidental and intentional ingestion of antibacterial NMs is on the rise within the human population, assessment of their impact on gut resident bacteria using in vitro methods has been overlooked. The present study has found CuO NMs to induce an antibacterial response in both a concentration- and time-dependent manner against three gut-relevant bacteria: *E. coli* K12, *E. faecalis*, and *L. casei.* These findings align with previous in vitro studies; for example, Chatterjee et al. (2012) [72] observed Cu NMs have a bactericidal effect on *E. coli* K12 at a concentration of 7.5 µg/mL, with cell viability decreasing as NM concentration increased. Sizentsov et al. (2018) [26] also found Cu NMs to inhibit the growth of *Lactobacillus*, with increased toxicity at higher concentrations (30 µg/mL). In vivo studies have also found Cu NMs to induce dysbiosis of the gut microbiota. For example, Merrifield et al. (2013) [24] found alterations in bacterial community structure with disruption to commensal bacteria such as *Cetobacterium somerae* in zebrafish following 14 days of dietary intake of Cu NMs (500 mg/kg). Similarly, Cu NMs have been found to induce changes to the microbiome of broiler chickens following oral exposure for 28 days at a dosage of 1.7 mg/kg. Notably decreasing the presence of *Lactobacillus* by 9% when compared with the control [73]. In contrast to the current data, some in vitro studies have observed CuO NMs to have no antibacterial activity against *lactobacillus* even after prolonged exposure to a concentration of 30 µg/mL [26]. Discrepancies between studies are likely to emerge due to differences in the PC properties of the NMs being tested and their experimental design (e.g., NM concentration, media type, time point, and assay employed to assess toxicity).

The bacterial strains used in the current study varied in their sensitivity to CuO NM toxicity. This bacterial-dependent effect is likely due to the varying biochemical features and mechanisms of defence these microorganisms possess, namely surface charge, membrane permeability, and cell wall composition [74]. The current data presents Gram-negative *E. coli* K12 to be more sensitive to CuO NM toxicity when compared with the Gram-positive strains (*E. faecalis* and *L. casei*). This result is in concordance with the literature, with Ayaz Ahmed and Anbazhagan (2017) [63] finding CuO NMs to significantly inhibit the growth of Gram-negative *P. aeruginosa* and *E. coli*, whilst having a limited antibacterial action against Gram-positive *S. aureus*. The thick peptidoglycan layer of Gram-positive bacteria could limit the uptake of NMs and their toxic ions and thus may protect against NM toxicity [75]. Conversely, the anionic surface domains of Gram-negative bacteria may allow for the binding of NMs [76]. Other studies have found the sensitivity of the bacteria against CuO NMs to be strain-specific and not solely dependent on the Gram type of the bacteria [22,72,74].

Overall, due to the conflicting nature of research assessing antibacterial NMs against gut bacteria, further studies are essential. To further determine if CuO NMs affect gut bacteria, studies should employ an array of (commensal) bacterial strains, e.g., *Lactobacillus* spp., *Bifidobacterium* spp., *Porphyromonas* spp., and *Clostridium* spp., which are resident in the gut. Additionally, future research should consider characterising the surface change of the bacteria by quantifying the ζ-potential via DLS as previously conducted by Halder et al. (2015) [77] or by fusing charged ligands to the bacteria to give them an artificial charge as previously conducted by Feng et al. (2015) [56]. Visualising and quantifying NM interactions with bacterial cells should also be prioritised in the future, as existing studies demonstrate that NMs will interact with the cell surface; for example, Sirelkhatim et al. (2015) [52] employed scanning electron microscopy (SEM) to visualise the interactions between ZnO NMs and *E. coli*, observing morphological changes in the bacteria. This will aid in determining if the biochemical features of the bacteria can influence their susceptibility to antibacterial NM.

### 4.2. Sensitivity of In Vitro Approaches

In vitro approaches are commonly used to assess the antibacterial effects of NMs, although most studies focus on pathogenic bacteria. Plate counts and diffusion assays are regularly employed to assess antibacterial properties [22]. The present study used a battery of assays that were identified to vary concerning their sensitivity. The plate counts conducted in the current study only found CuO NMs to have a significant antibacterial impact on *E. coli* K12 at all concentrations assessed. Surprisingly, the diffusion assays within the current study lacked sensitivity. Disc and well diffusion assays are commonly used assays and have previously been successfully used to assess the antibacterial activity of NMs [22,78]. The ineffectual response observed in this study may be due to the limited mobility of CuO NMs in agar and their inability to diffuse, which impedes their interaction with the bacterial cells [79]. Previous work assessing the antibacterial properties of CuO NMs has also shown limited results using this approach. For example, Masri et al. (2022) [22] found diffusion assays to detect CuO NM toxicity only at the highest concentrations tested (200 µg/mL), with small zones of inhibition (ZOI) measured. Similarly, in another study, *Lactobacillus* and *Enterococcus* strains exposed to CuO NM did not induce any inhibition of growth via the well diffusion assay [26].

OD measurements were successfully used in the present study to investigate the impact of CuO NMs on bacterial cell viability. A recent study also found the cell viability of *E. coli* and *B. subtilis* to be significantly reduced by CuO NMs (200 μg/mL) at 4, 8, and 24 h via OD readings [22]. A particular advantage of OD measurements is that they allow for the high throughput, continual assessment of bacterial growth inhibition over time. This is emphasised by the work of Dadi et al. (2019) [67], in which OD measurements were used to determine that *E. coli*, *S. aureus*, and *P. aeruginosa* are most sensitive to CuO NMs during the exponential growth phase. It is worth noting when using OD measurements, appropriate controls are essential, as the optical properties of NM are known to interfere with results [68]. Lastly, the live/dead fluorescent imaging approach found the viability of all bacterial models was reduced by CuO NM exposure, suggesting *E. coli* K12 was the most sensitive strain, which is in agreement with the other assays used. Similar results have also been observed by Masri et al. (2022) [22] using the same live/dead staining method. In the future, it is therefore recommended that plate counts and OD readings be prioritised when assessing the antibacterial activity of NMs to streamline investigations, as they allow a rapid, efficient, and cheap assessment of toxicity.

### 4.3. Physiochemical Properties of CuO NMs

The PC properties of NMs (e.g., size, shape, charge, solubility, surface area) influence their interactions with cells and therefore their antibacterial activity [15,80]. As a consequence, the PC properties of NMs are characterised in parallel to assessing their toxicity to identify what PC properties confer toxicity. Furthermore, there is evidence that information provided by suppliers on NM PC properties is not always accurate [81,82,83] and thus it is prudent to perform an independent characterisation of the PC properties of NMs. The PC properties of the CuO NMs being investigated in our study have been extensively characterised previously [64,65,66], and so only limited characterisation of the PC properties of the NMs was performed in this study to avoid the duplication of existing research.

The size of NMs directly correlates to their reactivity, with toxicity typically increasing with decreasing particle size [15]. Azam et al. (2012) [84] found the antibacterial activity of CuO NMs to be dependent on particle size, with smaller particles (20 nm) inducing the greatest toxicity against both Gram-positive and Gram-negative bacterial strains. The primary particle size of the CuO NMs investigated in this study is ~10–20 nm [64,65]. However, the hydrodynamic diameter of the CuO NMs was found to be ~200 nm when suspended in water, which suggests that the CuO NMs were agglomerated. This finding is in agreement with other studies in the published literature that have used the same CuO NMs [22,85]. The agglomeration of NMs can influence their interaction with bacterial cells and therefore their toxic potency, and so future studies should conduct studies to visualise the interaction of CuO NMs with bacterial cells, and if deemed necessary, the dispersion protocol used could be modified to reduce the likelihood of NM agglomeration.

CuO NM dissolution is also known to influence their toxicity [86,87,88]. The solubility of the exact CuO NMs tested in our study has been investigated previously, with evidence that these NMs are highly soluble [64,66] This suggests that ions rather than particle effects may drive the toxicity of the CuO NMs to bacteria. Indeed, there is evidence that Cu ions can be absorbed by bacterial cells and disrupt many biochemical functions [89,90] and promote intracellular ROS production, which supports our hypothesis [86]. Thus, future studies should therefore evaluate ion release from CuO NMs as it is essential to their antibacterial function using inductively coupled plasma-mass spectrometry (ICP-MS) or ICP-OES to monitor dissolution over time, as this will help distinguish between particle or ion effects [64,91]. In addition, an ionic control, such as copper sulphate, could be included in future experiments to help distinguish between particle- and ion-mediated antibacterial effects.

### 4.4. CuO NM-Mediated Intracellular ROS Production

It is well established in the literature that NMs mediate ROS production and stimulate oxidative stress [63,86]. The formation of ROS is widely hypothesised to be a key antibacterial mechanism of CuO NMs [13]. Within the present study, there is evidence that CuO NMs generate ROS in all bacterial strains, supporting this proposed MOA. Previous studies employing fluorescent imaging with DCFH-DA probes have found similar results, observing increased ROS production in *S. aureus, E. coli*, and *B. subtilis* following CuO NM exposure [63,92]. Given the growing evidence implicating ROS in NM-mediated antibacterial activity, future studies should consider incorporating the DCFH-DA assay to visualise and quantify ROS generation following NM exposure as a critical endpoint in NM toxicity evaluations.

### 4.5. Conclusions and Future Prospects

In conclusion, the obtained results suggest that CuO NMs induce a time- and concentration-dependent antibacterial response against gut-relevant bacteria. Among the assessed strains, Gram-negative *E. coli* K12 exhibited the greatest sensitivity to CuO NM toxicity. Following a mechanistic assessment of intracellular ROS production via fluorescent imaging using the DCFH-DA probe, all bacterial strains treated with CuO NM demonstrated an increase in ROS production.

Gaining a better understanding of the impact that ingested NMs have on the gut microbiota will help inform potential adverse health outcomes. Indeed, it has been proposed that the assessment of microbiota dysbiosis is a key component of the hazard assessment of ingested NMs [93]. Whilst a reliance has been placed on using in vivo models to assess this hazard outcome, to date, it is advised that in the future in vitro models be used to a greater extent. The use of a combination of assays provides a robust approach to assess the antibacterial properties of NMs in vitro, and it is therefore recommended that OD measurements and plate counts be prioritised in the future when assessing the antibacterial activity of NMs in vitro, as they are consistently identified as being the most sensitive assays. Our study, and those in the published literature, have considered the detrimental impact of CuO NMs on individual strains of bacteria in vitro [22,26]. Future studies should also consider assessing the antibacterial impact of NMs on communities of relevant gut bacteria in vitro to improve the physiological relevance of the test model. For example, a recent study employed an in vitro human gut simulator containing gut microbiota samples from healthy male volunteers to assess the impact of TiO_2_ and Ag NMs on bacterial communities. TiO_2_ NMs exhibited limited reductions in bacteria density, whereas antibacterial Ag NMs reduced community density by 62% [94]. Agans et al. (2019) [94] also observed no alteration in the community diversity after NM exposure, which may be an indication that although bacterial viability is reduced, gut dysbiosis does not occur. This approach would be beneficial to future assessment, as it allows the interplay between the bacterial strains to be evaluated and tracks the potential recovery of the community following NM exposure. Furthermore, it is prudent for future work to evaluate the antibacterial properties of NM under anaerobic conditions when assessing their impact on the gut microbiota to allow for a more physiologically relevant assessment of toxicity. Indeed, Xiu et al. (2012) [34] observed Ag NMs to be significantly more toxic when studies were conducted in the presence of oxygen.

## Figures and Tables

**Table 1:** Percentage reduction in OD following CuO NM treatment compared to the control at 24 h post-exposure.

Bacteria	CuO NM (100 µg/mL)	CuO NM (200 µg/mL)	CuO NM (300 µg/mL)
*E. coli* K12	9%	46%	66%
*E. faecalis*	43%	47%	50%
*L. casei*	33%	76%	100%

## Data Availability

The data presented in this study are available on request from the corresponding author.
